# Postoperative quality of life and nutritional outcomes following subtotal, proximal, or distal gastrectomy for upper gastric cancer: a retrospective cohort study

**DOI:** 10.1007/s00595-026-03337-6

**Published:** 2026-06-03

**Authors:** Keishi Okubo, Takaaki Arigami, Masataka Shimonosono, Daisuke Matsushita, Ken Sasaki, Masahiro Noda, Kenji Baba, Yota Kawasaki, Takao Ohtsuka

**Affiliations:** Department of Digestive Surgery, Kagoshima University Graduate School of Medical and Dental Sciences, 8-35-1 Sakuragaoka, Kagoshima, 890-8520 Japan

**Keywords:** Subtotal gastrectomy, Proximal gastrectomy, Distal gastrectomy, Upper gastric cancer, Postoperative quality of life, Nutritional outcomes

## Abstract

**Purpose:**

The optimal surgical approach for upper gastric cancer remains controversial, particularly regarding the postoperative quality of life (QOL) and nutritional outcomes. This study compared the outcomes of subtotal gastrectomy (STG), proximal gastrectomy (PG), and distal gastrectomy (DG).

**Methods:**

We retrospectively analyzed 94 patients who underwent gastrectomy for upper gastric cancer (DG: *n* = 39, PG: *n* = 33, STG: *n* = 22). The quality of life (QOL) was assessed using the Postgastrectomy Syndrome Assessment Scale (PGSAS-45) 12 months postoperatively. The nutritional status was evaluated based on changes in body weight.

**Results:**

The baseline clinicopathological factors and TNM stages were comparable among the three cohorts. PG was associated with significantly worse reflux, dyspepsia, and meal-related complaints than STG and DG. The STG showed fewer dumping-related symptoms than the PG. No significant differences in overall nutritional status were observed between the STG and DG groups, whereas the PG group tended to be associated with less favorable nutritional outcomes. These findings were consistent with the responder analyses.

**Conclusions:**

In this retrospective cohort study, STG was associated with a more favorable postoperative symptom profile than PG and showed postoperative outcomes comparable to those of DG.

**Supplementary Information:**

The online version contains supplementary material available at https://doi.org/10.1007/s00595-026-03337-6.

## Introduction

Gastric cancer is a major global health burden. It is particularly prevalent in East Asia, where surgical resection with adequate lymphadenectomy is the cornerstone of curative treatment [1]. Total gastrectomy (TG) has long been considered the standard approach for upper-third gastric cancer; however, it inevitably eliminates gastric reservoir function, resulting in impaired postoperative nutrition and quality of life (QOL) [2].

With increasing attention to survival and functional recovery, function-preserving strategies have attracted attention. Subtotal gastrectomy (STG), which retains a small remnant stomach and partial reservoir capacity, has been proposed as a feasible alternative for selected upper gastric cancers when negative proximal margins and adequate lymphadenectomy are achieved. According to previous retrospective studies, STG offers favorable short-term outcomes, acceptable oncological safety, and superior nutritional maintenance compared with TG [3–7]. Furthermore, meta-analyses have suggested that STG reduces long-term symptom burden and postoperative complications compared with TG [8–11]. However, whether STG provides comparable quality of life (QOL) benefits to those of distal gastrectomy (DG) and proximal gastrectomy (PG) remains unknown.

The Postgastrectomy Syndrome Assessment Scale-45 (PGSAS-45) is a validated questionnaire designed to comprehensively evaluate symptoms, living status, and QOL after gastrectomy [12]. This scale integrates 22 original items with 23 items from the Short-Form Health Survey (SF-8) and Gastrointestinal Symptom Rating Scale [13–16]. Total gastrectomy has traditionally been considered the standard surgical procedure for upper-third gastric cancer. However, several previous studies have demonstrated that subtotal gastrectomy provides better postoperative quality of life and nutritional outcomes compared with total gastrectomy because it preserves a small gastric reservoir and a partial physiological function. According to large-scale analyses using PGSAS-45, small remnant DG outperforms TG in multiple domains, including reflux, meal-related distress, work ability, QOL dissatisfaction, and postoperative body weight change [3, 5]. These findings underscore the clinical importance of preserving gastric reservoir function and physiological passage. Most previous studies only compared two procedures (e.g., STG vs. TG or PG vs. TG). Direct three-way comparisons among DG, PG, and STG remain limited. These procedures substantially differ in gastric preservation, motility, reflux physiology, and nutritional outcomes. Because of the lack of comprehensive comparative data, surgeons face challenges in selecting the optimal procedure for upper gastric cancer based on oncological safety and long-term QOL. To date, although STG yields a smaller remnant stomach than DG, no studies have clarified whether STG is inferior to DG in terms of postoperative QOL or nutritional outcomes. By directly comparing STG and DG with Roux-en-Y(RY) reconstruction in the present study, we aimed to evaluate whether STG is an acceptable function-preserving procedure. If the postoperative QOL and nutritional status after STG are not inferior to those after DG, this would support the clinical utility of STG as a function-preserving surgical option even with a smaller remnant stomach.

To address this gap, this retrospective study compared the postoperative QOL and nutritional outcomes among patients who underwent DG, PG, and STG, using the PGSAS-45 and body weight ratio (BWR) as markers of long-term physiological function. This study aimed to provide clinically meaningful evidence to guide function-preserving surgical decision-making in upper gastric cancer by integrating balanced clinicopathological backgrounds with standardized symptom evaluation.

## Materials and methods

### Patients

We retrospectively reviewed the records of 414 patients who underwent DG, PG, or STG for upper gastric cancer at Kagoshima University Hospital between January 2011 and December 2024. Considering that reconstruction methods may influence the surgical outcomes and QOL, only patients who underwent PG with double-tract (DT) reconstruction and DG with RY were included. Among the 195 eligible patients, 94 who completed the PGSAS-45 questionnaire 12 months postoperatively were analyzed. Patients who could not complete the questionnaire were excluded.

**Fig. 1:**
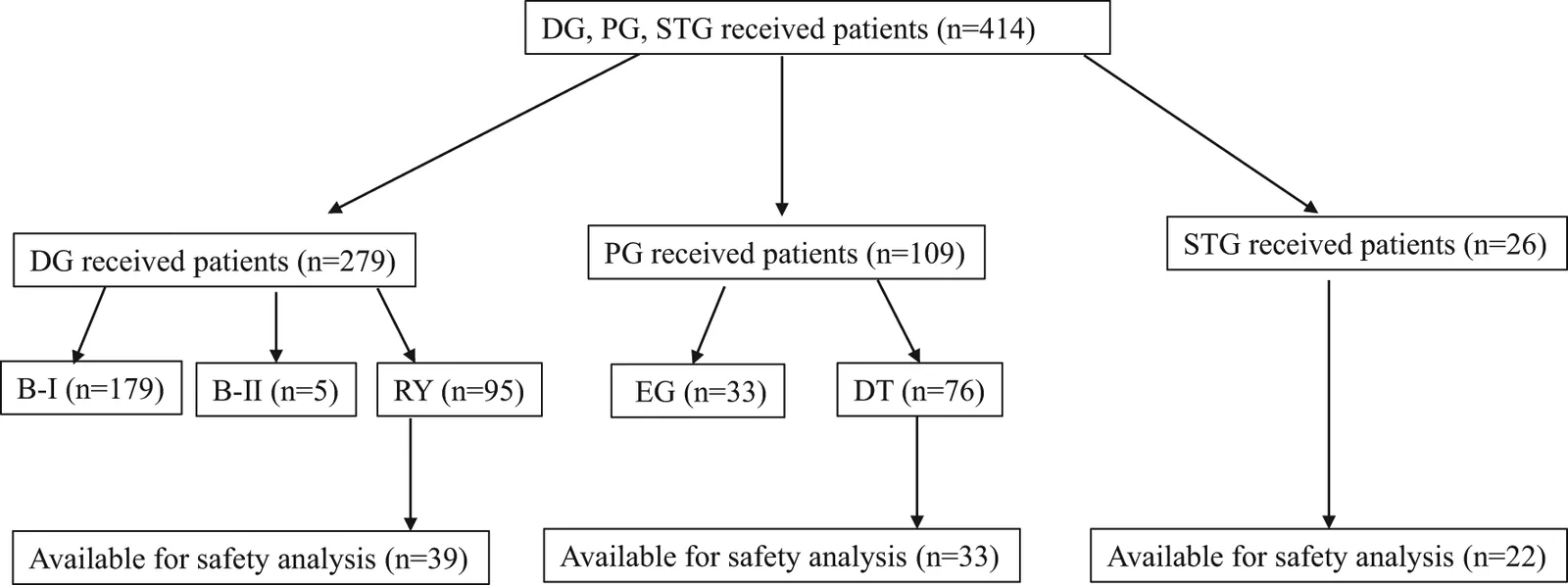
Patient flow diagram. Abbreviations: B-Ⅰ, Billroth Ⅰ reconstruction; B-Ⅱ, Billroth Ⅱ reconstruction; DG, distal gastrectomy; DT, double tract reconstruction; EG, esophagogastrostomy; PG, proximal gastrectomy; RY, Roux-en-Y reconstruction; STG, subtotal gastrectomy

This study was approved by the Institutional Review Board of Kagoshima University (approval number: 230068). Informed consent was obtained using an opt-out approach. Patients were categorized into three groups: DG (*n* = 39), PG (*n* = 33), and STG (*n* = 22) (Fig. 1).

## Surgical procedures

The indications for STG were based on the tumor location and lymph node status. Patients with swelling in nodes No. 2, 4sa, or 4sb or tumors involving the greater curvature were excluded. Tumor extent was assessed using preoperative esophagogastroduodenoscopy. The transection line was selected to ensure a proximal margin of ≥ 2 cm, which was intraoperatively confirmed by endoscopy and frozen-section analysis. STG resulted in a small remnant stomach with ≤ 2 cm of the lesser curvature from the gastric stump to the esophagogastric junction. Regional lymphadenectomy was performed according to the Japanese Gastric Cancer Treatment Guidelines [17]. RY was used for the STG and DG. PG was performed when the distal remnant stomach was approximately half its original size, followed by DT via the antecolic route.

## Assessment of postoperative QOL

QOL was evaluated using the PGSAS-45 questionnaire, which includes 45 items (8 items from the SF-8, 15 items from the Gastrointestinal Symptom Rating Scale, and 22 original items developed by the Japanese Postgastrectomy Syndrome Working Party). Responses were recorded using five- or seven-point Likert scale. The main outcome measures (MOMs) comprised seven symptom scales (reflux, abdominal pain, meal-related distress, indigestion, diarrhea, constipation, and dumping), four living status scales (food intake per meal, need for additional food, quality of ingestion, and work ability), and one quality of life (QOL) scale (dissatisfaction with daily life). The questionnaires were completed 12 months postoperatively.

## Evaluation of nutritional status

BWR was calculated as follows: postoperative body weight divided by preoperative body weight. The prognostic nutritional index (PNI) was calculated as follows: 10 × serum albumin (g/dL) + 0.005 × total lymphocyte count (per mm^3^) [18]. The PNI ratio was calculated as the postoperative PNI divided by the preoperative PNI.

### Statistical analysis

Values are presented as the mean ± SD or median and range. Two-group differences in the mean values were analyzed using an unpaired *t*-test, and multiple-group differences were analyzed using a one-way analysis of variance (ANOVA). The Bonferroni-Dunn test was used when ANOVA yielded a *P* value of < 0.1. Generally, a *P* value of < 0.05 on the *t*-test or ANOVA was considered to be statistically significant. For Bonferroni/Dunn multiple comparisons, a *P* value of < 0.05 divided by the number of comparisons was considered statistically significant. When the *P* value was less than twice the significance level, Cohen’s *d* was calculated to determine the effect size. The value of Cohen’s *d* reflects the effect of each causal variable, with 0.2 to < 0.5 denoting a small but clinically meaningful effect and 0.5 to < 0.8 and ≥ 0.8 denoting medium and large effects, respectively. All statistical analyses were performed using the JMP 19.0 software program (SAS Institute Inc., Cary, NC, USA).

## Results

### Patient characteristics

The baseline characteristics of the patients are summarized in Table 1. We retrospectively analyzed data from 95 patients who underwent R0 resection. The operation time was significantly shorter in the DG group, whereas intraoperative blood loss was the lowest in the STG group (*P* < 0.01 and *P* = 0.03, respectively). Age, tumor stage (pT, pN, and pStage), histological type, postoperative complications, and adjuvant chemotherapy use did not differ significantly among the three groups, indicating comparable oncological backgrounds. In the STG group, the median distance between the esophagogastric junction (EGJ) and the tumor was 50 mm (range:35–60 mm), and no cases of tumor invasion into the EGJ were observed. Severe postoperative complications (Clavien–Dindo grade ≥ 3) occurred in six patients, and no patient required readmission after discharge.

**Table 1:** Clinicopathological Clinicopathological characteristics of patients

		DG (*n*=39)	PG (*n*=33)	STG (*n*=22)	*P*-value
Gender					0.03
	Male	25	30	16	
	Female	14	3	6	
Median age, years (range)		69 (36-88)	65 (43-87)	70 (33-85)	0.47
Tumor diameter (mm) median (range)		40.1 (5-85)	30.1 (8-120)	36.5 (11-81)	0.08
Location, U/M//L		0 / 23 / 16	29 / 4 / 0	4 / 18 /0	<0.01
Circumference					0.19
	Less	17	19	10	
	Ant	7	2	1	
	Post	5	9	5	
	Gre	7	3	6	
	Circ	3	0	0	
pT factor					0.33
	T1-2	30	29	16	
	T3-4	9	4	6	
pN factor					0.89
	N0	32	27	17	
	N1-3	7	6	5	
pStage					0.43
	I	26	27	14	
	II	8	5	6	
	Ⅲ	5	1	2	
Histopathological type					0.40
	Differentiated	19	21	11	
	Undifferentiated	20	12	11	
Mean operation time (min) *		406.6±14.1	478.2±15.3	430.9±18.8	<0.01
Mean blood loss (ml)*		160.5±38.0	162.4±41.3	105.5±50.6	0.03
Lymph Node dissection					<0.01
	D1+	22	31	12	
	D2	17	2	10	
Number of lymph node dissection		31 (3-40)	22 (4-41)	31 (4-56)	0.07
Postoperative complication (CD≦2)		7	8	4	0.77
Adjuvant chemotherapy		13	5	4	0.15
Recurrence		3	1	0	0.36

CD : Clavien - Dindo, DG : Distal gastrectomy, PG : Proximal gastrectomy, STG : Subtotal gastrectomy

*Mean ± SD

## QOL assessment by PGSAS-45

**Fig. 2:**
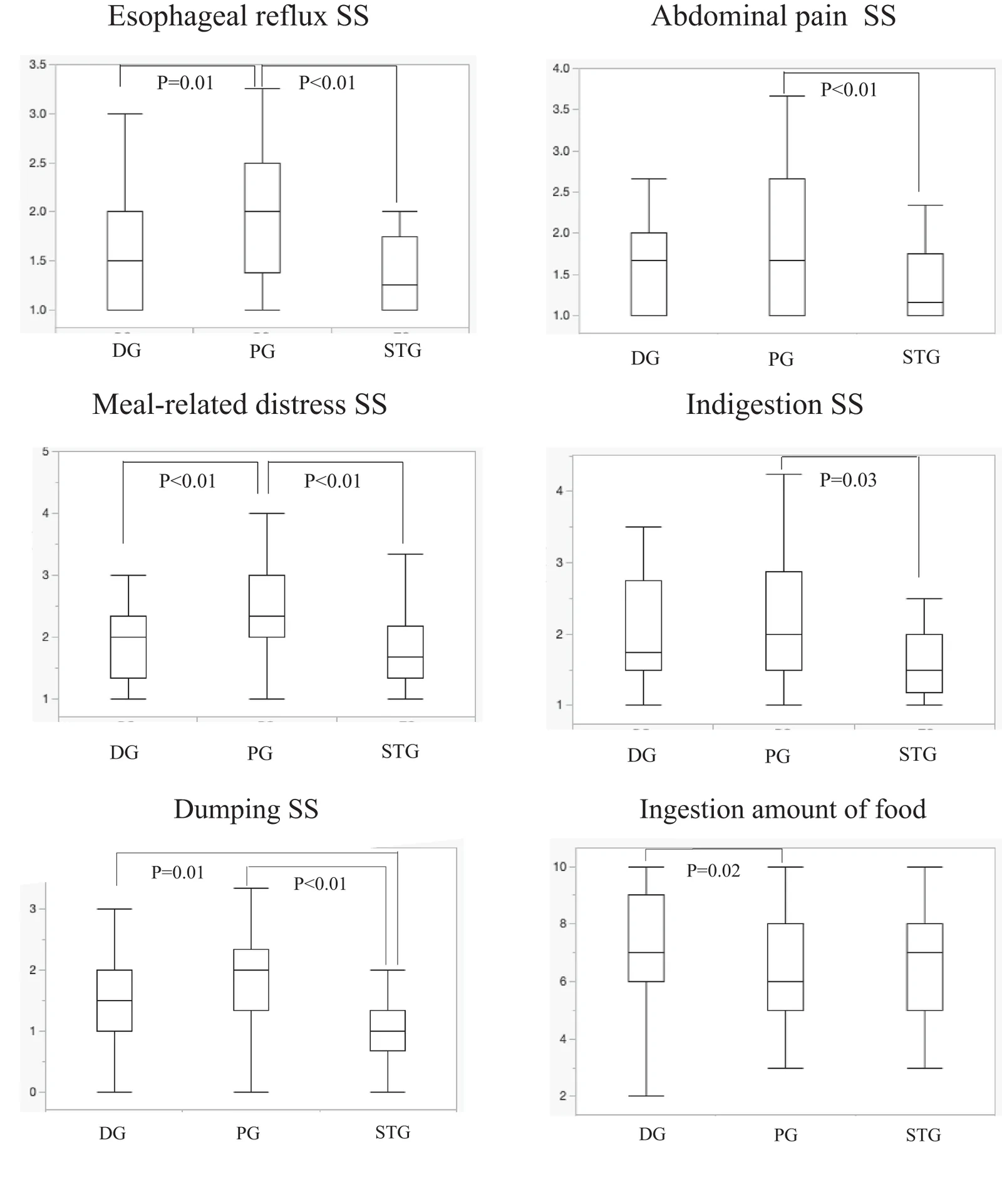
PGSAS-45 at 12 months postoperatively in DG, PG, and STG groups. Abbreviations: PGSAS-45, Postgastrectomy Syndrome Assessment Scale-45; DG, distal gastrectomy; PG, proximal gastrectomy; STG, subtotal gastrectomy

The results of the unadjusted group comparisons of the PGSAS MOMs at 12 months postoperatively are presented in Table 2. Esophageal reflux scores were significantly better in the STG and DG groups than in the PG group (DG vs. PG: *P* = 0.01; STG vs. PG: *P* < 0.01). Meanwhile, the abdominal pain scores were better in the STG group than in the PG group (PG vs. STG: *P* < 0.01). Meal-related distress was lower in the STG and DG groups than in the PG group (DG vs. PG: *P* < 0.01; STG vs. PG: *P* < 0.01). Indigestion scores were higher in the STG group than in the PG group (P *=* 0.03). The dumping scores were the lowest in the STG group, with significant differences compared to the DG (*P* = 0.01) and PG (*P* < 0.01) groups. The food intake per meal was higher in the DG group than in the PG group (*P* = 0.02) (Fig. 2).

**Table 2:** The main outcomes measures at 12 months after gastrectomy

MOMs	DG	PG	STG	ANOVA *P* value	Tukey	*P* Value	Cohen’s d value
Esophageal reflux SS	1.53±0.57	1.94±0.69	1.36±0.38	<0.01	sTG vs. PG	<0.01	1.48
					sTG vs. DG	0.51	
					PG vs. DG	<0.01	0.72
Abdominal pain SS	1.58±0.52	1.83±0.75	1.40±0.50	0.03	sTG vs. PG	<0.01	0.85
					sTG vs. DG	0.51	
					PG vs. DG	0.20	
Meal-related distress SS	1.86±0.64	2.58±0.77	1.89±0.69	<0.01	sTG vs. PG	<0.01	1.00
					sTG vs. DG	0.99	
					PG vs. DG	<0.01	1.05
Indigestion SS	2.05±0.76	2.12±0.85	1.59±0.50	0.03	sTG vs. PG	0.03	1.04
					sTG vs. DG	0.06	
					PG vs. DG	0.91	
Diarrhea SS	1.95±0.83	1.87±0.62	1.77±0.89	0.68			
Constipation SS	2.22±1.07	2.26±0.92	1.87±1.00	0.34			
Dumping SS	1.65±0.83	1.83±0.77	1.03±0.68	<0.01	sTG vs. PG	<0.01	1.22
					sTG vs. DG	0.01	0.95
					PG vs. DG	0.62	
Ingestion amount of food	7.34±1.82	6.12±1.70	6.63±2.01	0.02	sTG vs. PG	0.56	
					sTG vs. DG	0.32	
					PG vs. DG	0.02	0.72
Necessity for additional food	1.92±0.82	2.30±0.72	2.00±0.87	0.16			
Quality of ingestion SS	3.71±0.84	3.46±1.07	3.77±0.83	0.39			

MOMs: main outcome measures SS: subscale, DG : Distal gastrectomy, PG : Proximal gastrectomy, STG : Subtotal gastrectomy

### Changes in body weight and nutritional indicators

**Fig. 3:**
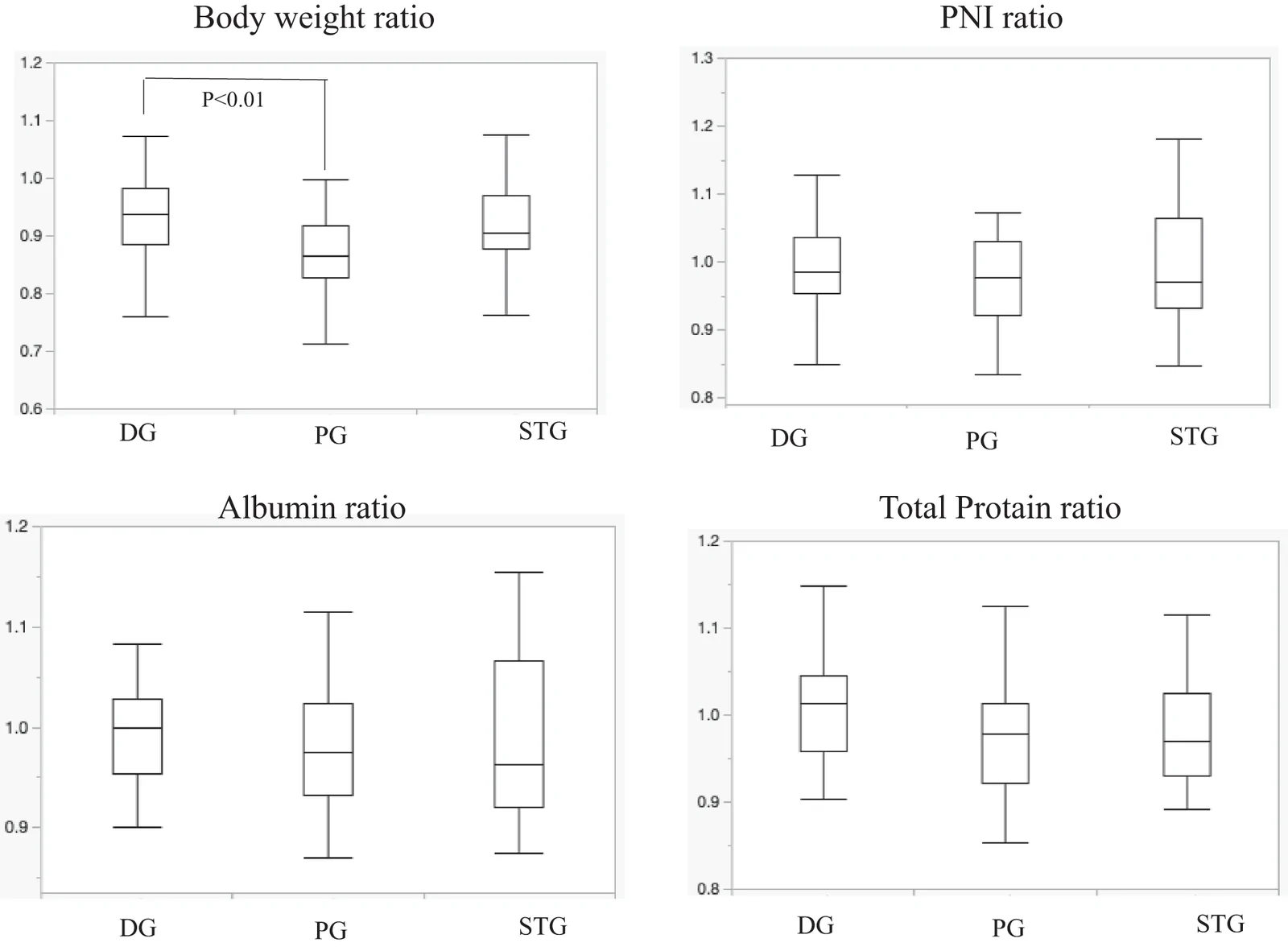
Changes in body weight and nutritional indicators at 12 months postoperatively

The BWR at 12 months postoperatively is shown in Fig. 3. The body weight ratio differed significantly among the three groups (*P* = 0.03). The DG group maintained a significantly higher BWR than the PG group (*P* < 0.01). However, no significant differences were observed in the PNI, total protein, or albumin ratios among the three groups (all *P* > 0.05) (Table 3).

**Table 3:** Evaluation of body weight ratio and nutritional status at 12 months after gastrectomy

Parameters	DG	PG	STG	*P*-value
Body weight ratio	0.93±0.10	0.86±0.08	0.91±0.07	0.03
Hemoglobin levels	12.3	12.8	12.6	0.31
PNI ratio	1.01±0.10	0.97±0.06	0.98±0.06	0.17
Albumin ratio	1.01±0.09	0.98±0.06	0.98±0.08	0.23
Total protein ratio	1.02±0.11	0.97±0.06	0.98±0.06	0.11

PNI: prognostic nutritional index, DG : Distal gastrectomy, PG : Proximal gastrectomy, STG : Subtotal gastrectomy

## Discussion

Postgastrectomy symptoms, including reflux, abdominal discomfort, and dumping, significantly affect the postoperative QOL. The PGSAS-45 questionnaire, developed by the Japanese Postgastrectomy Syndrome Working Party, is a validated tool for evaluating symptoms, living status, and QOL [19–22]. Recent studies have applied the PGSAS-45 to assess functional outcomes after various gastrectomy procedures, including functional-preserving approaches (e.g., local resection based on sentinel node navigation surgery), which demonstrated a superior QOL compared with DG [23]. In the present study, PGSAS-45 was used to compare STG, PG, and DG in patients with upper-tract gastric cancer.

Previous reports of the PGSAS NEXT study revealed that both a double-tract procedure and esophagogastrostomy were widely selected for reconstruction after PG [24]. STG, which preserves a small remnant stomach, was first described by Jiang et al. in 2011 [25]. The Japanese Gastric Cancer Treatment Guidelines weakly recommend preserving a minimal remnant stomach when adequate resection margins can be secured [17]. Although there are concerns regarding lymphadenectomy and proximal margins in advanced disease, accumulating evidence suggests that STG is oncologically feasible for selected upper-third gastric cancers, particularly cT1N0 tumors, when intraoperative margin confirmation and lymph node assessment are performed [26, 27]. Moreover, the functional benefits of gastric preservation have been well documented. According to previous studies, STG with a small remnant stomach yields a better QOL, less weight loss, and improved living function compared with TG [28, 29]. Soneda et al. also observed favorable body weight and muscle mass changes with STG compared with PG [30]. However, direct comparisons among STG, DG, and PG have been limited.

Our data were obtained from a single institution, where surgical procedures and treatment strategies were relatively standardized. Therefore, the potential bias related to variations in surgical technique and perioperative management may be limited. In addition, postoperative outcomes were evaluated 12 months after surgery, which has been reported in previous studies to be the time point at which postgastrectomy symptoms become most apparent and are considered to best reflect patients’ long-term quality of life. However, changes in the postoperative symptoms and nutritional status over time could not be assessed. Future studies with longitudinal evaluations are needed to better understand the temporal changes in postoperative functional outcomes. The findings of the present study indicate that STG provides a superior postoperative QOL and nutritional stability compared with PG with DT reconstruction and comparable outcomes to DG. Symptom domains, including reflux, abdominal pain, and dumping, were significantly better in STG than in PG with DT reconstruction. Both STG and DG maintained higher BWR than PG, emphasizing the physiological advantage of gastric reservoir preservation. Interestingly, STG showed lower dumping scores than DG, despite the latter having a larger remnant stomach and higher meal intake. We believe the difference in the clinical symptoms was from some reasons. The remnant stomach volume, gastroparesis and deformation after gastrectomy are associated with QOL and several symptoms. Remnant stomach volume is important for retention to prevent dumping syndrome. The better dumping scores observed after STG cannot be explained solely by the remnant stomach volume. Dumping syndrome is primarily related to rapid gastric emptying and abrupt delivery of nutrients into the small intestine rather than the absolute size of the gastric remnant. In STG, extensive transection of the gastric nerves may impair peristaltic activity of the remnant stomach more than in DG. This impaired motility could delay gastric emptying and reduce the amount of food ingested per meal, which may consequently contribute to the lower incidence of dumping symptoms observed in the STG group.

These results have clinical implications, particularly in elderly patients. In a study by Terayama et al., laparoscopic function-preserving gastrectomy, including STG, was safe and beneficial for older patients, improving nutritional parameters and reducing skeletal muscle loss [31]. Considering the complexity of reconstruction and longer operating time in PG, STG may represent a practical option for elderly individuals, potentially reducing postoperative complications (e.g., pneumonia), which significantly affect prognosis. Consequently, invasive STG can offer a balance between oncological safety and functional preservation, especially in frail populations.

Taken together, STG appears to provide favorable long-term symptom control and nutritional maintenance when negative margins and adequate lymphadenectomy are achieved. Moreover, it could offer benefits by preserving the secretion of ghrelin (which is secreted from the fundic glands of the stomach and has pleiotropic functions, including appetite stimulation, bowel movement regulation, and nutrient absorption). Even though only 30% of the stomach is preserved, STG has also been shown to preserve appetite and cause considerably less weight loss than TG [32, 33]. This may be attributed to the fact that STG preserves the ghrelin secretion region by preserving the greater curvature of fornix.

The present study has several limitations that should be acknowledged. This preliminary study was retrospective, performed at a single center, and based on a small sample size (*n* = 94), which may have introduced some bias. As only patients who completed the PGSAS-45 questionnaire at 12 months were included in the analysis, responder bias could not be completely excluded. In particular, differences in the pathological stage and the use of adjuvant chemotherapy between responders and non-responders may reflect differences in disease severity (Supplementary Table 1). Moreover, variations in the reconstruction techniques and surgeon preferences could affect the outcomes. Therefore, future multicenter prospective studies with standardized reconstruction and objective functional assessments are required to confirm these findings. It should be noted that the findings regarding proximal gastrectomy in the present study specifically reflect proximal gastrectomy with DT reconstruction, and the observed differences may be partly influenced by the reconstruction method. In addition, the reconstruction methods differed among the surgical groups. Therefore, the effects of gastrectomy extent could not be completely separated from those of reconstruction. In addition, although postoperative nutritional status was evaluated using body weight ratio and hemoglobin levels, other nutritional parameters such as vitamin B12 levels, iron indices, and skeletal muscle mass were not evaluated in this study. Therefore, the nutritional assessment in this study may not fully reflect all aspects of the postoperative nutritional status.

## Conclusion

In this cohort, STG was associated with more favorable PGSAS-45 scores at 12 months postoperatively. It was also associated with a relatively better postoperative nutritional status in patients with upper gastric cancer. Therefore, STG may be considered a potential function-preserving surgical option when adequate proximal margins and appropriate lymph node dissection are achieved. However, these results should be interpreted with caution, as they reflect associations observed in a selected cohort and may be influenced by differences in the reconstruction methods.

## Supplementary Information

Below is the link to the electronic supplementary material.

Supplementary Material 1
