# Supplementary material for: Postoperative quality of life and nutritional outcomes following subtotal, proximal, or distal gastrectomy for upper gastric cancer: a retrospective cohort study
Source: Surg Today. 2026 Jun 3;56(10):2149–57. doi: 10.1007/s00595-026-03337-6 (PMC13558340; doi:10.1007/s00595-026-03337-6)
Supplement: Supplementary file 1 [file 595_2026_3337_MOESM1_ESM.docx]

**Supplementary Table 1. Clinicopathological characteristics of patients**

|  |  | **responder**  **(n=94)** | **Non-responder**  **(n=101)** | **P-value** |
| --- | --- | --- | --- | --- |
| Gender | |  |  | 0.54 |
|  | Male | 71 | 81 |  |
|  | Female | 23 | 20 |  |
| Median age, years (range) | | 67 (36-88) | 70 (43-87) | 0.28 |
| BMI * | | 23.4±3.71 | 23.0±3.60 | 0.46 |
| pStage | |  |  | <0.01 |
| I,Ⅱ | | 86 | 70 |  |
| Ⅲ,Ⅳ | | 8 | 31 |  |
| Post operative complication (CD≦2) | | 19 | 17 | 0.52 |
| Adjuvant chemotherapy | | 22 | 42 | <0.01 |
| Recurrence status | | 11 | 18 | 0.21 |

BMI: Body Mass Index CD : Clavien - Dindo,

*Mean ± SD
